# Superior mesenteric artery ischemia: endovascular approach

**DOI:** 10.1186/1471-2318-11-S1-A43

**Published:** 2011-08-24

**Authors:** G Patrizi, M Fazi, L Fiengo, G Di Rocco, D Giannotti, F Pelle, F Frezzotti, R Giordano, L Venturini, A Redler, FM Salvatori

**Affiliations:** 1 Dipartimento di Scienze Chirurgiche, Policlinico Umberto I, Università di Roma“La Sapienza”, Rome, Italy; 2 Dipartimento di Scienze Radiologiche, Policlinico Umberto I, Università di Roma“La Sapienza”, Rome, Italy

## Background

Mesenteric chronic ischemia is an unfrequent pathology usually related to obstructive and stenosing atherosclerotic pathologies of one or more digestive arteries. Among these, the most frequently envolved and revascularised is the superior mesenteric artery. There is a relative prevalence among the female elderly.

Symptoms are often represented by the typical *angina abdominis* and weight loss. Diarrhea can occur and, without appropriate treatment, this condition can evolve into intestinal infarction.

Treatment can be carried out with traditional surgery or a more recent endovascular approach.

## Materials and methods

We present the case of a 65-year-old patient who was on dialytic treatment for chronic renal failure, and in the past 18 months he was undergoing peritoneal dialysis. The latest sessions had to be interrupted because of the onset of peritonitic symptoms. In the patient’s personal history, besides hypertension and dislipidemia, he referred total parathyroidectomy for secondary hyperparathyroidism.

In the last month, he was complaining of the onset of abdominal pain in the right iliac fossa after meals, with homolateral lumbar irradiation, and, more recently during dialysis, which had to be interrupted immediately.

Plain abdominal radiograms showed diffuse calcification of the aorta and its branches, especially in lateral projection, where remarkable calcifications of the celiac trunk and SMA were evident.

Ultrasonography and Doppler demonstrated a pre-occlusive stenosis at the origin of the SMA and angioMRI confirmed these findings.

Because of the poor general clinical conditions of the patient and to preserve the abdomen for a potential renal transplantation, the endovascular approach was considered the best option. A metallic self-expandable vascular (Wallstent) stent of 7mm x 3 cm was placed with a successful outcome.

## Results

Postoperative course was uneventful with complete regression of symptoms and follow-up controls at 3, 6, 12 and 24 months showed no restenosis. The patient after this period is still asymptomatic.

## Conclusions

Endovascular treatment is an effective therapeutic alternative to surgery for the treatment of chronic mesenteric ischemia.

